# Factors associated with satisfaction of Italian physicians: a cross-sectional study in Rome

**DOI:** 10.3389/fpubh.2025.1584483

**Published:** 2025-05-30

**Authors:** Giuseppe Furia, Antonio Vinci, Aurora Heidar Alizadeh, Martina Sapienza, Cosimo Savoia, Maria Grazia Tarsitano, Cristina Patrizi, Massimo Maurici, Giovanni Capelli, Rome OMCeO Group, Antonio Magi, Stefano De Lillo, Guido Coen Tirelli, Gianfranco Damiani

**Affiliations:** ^1^Department of Public Health and Infectious Disease, Sapienza Università di Roma, Rome, Italy; ^2^Directive Council of the Physicians and Dental Surgeons Board of the Province of Rome, Rome, Italy; ^3^Doctoral School of Nursing Sciences and Public Health, Università di Roma “Tor Vergata”, Rome, Italy; ^4^Department of Health Sciences and Public Health, Section of Hygiene, Università Cattolica del Sacro Cuore, Rome, Italy; ^5^Health Management, Istituto Nazionale Malattie Infettive Lazzaro Spallanzani IRCCS, Rome, Italy; ^6^Department of Human Sciences and Promotion of the Quality of Life, San Raffaele Roma Open University, Rome, Italy; ^7^Local Health District, Rome, Italy; ^8^Department of Biomedicine and Prevention, Università di Roma “Tor Vergata”, Rome, Italy; ^9^National Center for Disease Prevention and Health Promotion, Istituto Superiore di Sanità (ISS), Rome, Italy; ^10^Department of Woman and Child Health and Public Health, Fondazione Policlinico Universitario “A. Gemelli” IRCCS, Rome, Italy

**Keywords:** work-life balance, healthcare worker, burnout syndrome, professional well-being, job satisfaction

## Abstract

**Introduction:**

Healthcare workers’ (HCW) quality of life and job satisfaction are critical for their well-being and performance, influencing patient outcomes and reducing burnout. Burnout, linked to excessive workloads, night shifts, and low income, is a recognized issue among HCW, exacerbated during the COVID-19 pandemic. This study aimed to explore work-life balance and professional satisfaction among members of the Physicians and Dental Surgeons Board of Rome (OMCeO Rome).

**Methods:**

A cross-sectional survey was conducted from March to June 2023 among OMCeO Rome members. An anonymous, digitally designed questionnaire assessed sociodemographic and professional data, perceived stress, and overall satisfaction using a Likert scale. Data were analyzed descriptively, and logistic regression identified predictors of satisfaction.

**Results:**

The survey included 1,104 respondents, predominantly aged over 50. Satisfaction levels were polarized: 47.4% reported high satisfaction, while 49.4% expressed low satisfaction. Night shifts and income below €100,000/year were significantly associated with lower satisfaction (OR 1.9 and OR 3.9, respectively). General practitioners/primary care paediatricians reported the highest stress levels, while self-employed professionals showed the lowest stress and intention to quit.

**Discussion:**

Work-life balance challenges, including night shifts and inadequate income, strongly influence HCW job satisfaction. Addressing these factors through administrative support, reduced workloads, and targeted interventions could mitigate burnout and improve care quality. Further studies should explore systemic and individual strategies to enhance HCW well-being and professional sustainability.

## 1 Introduction

The quality of life and job satisfaction of healthcare workers (HCW) are two fundamental, deeply connected dimensions that participate in determining both personal and community psychophysical well-being. According to scientific literature, the work-life balance quality of HCW has a strong impact on various aspects of their professional practice that, ultimately, influences the health outcomes of their patients (1). The establishment of a trusting doctor-patient relationship, notoriously crucial to the effectiveness of the prescribed therapy and eliciting compliance, as well as user satisfaction, may be heavily affected (2–4). Therefore, by pursuing a better work-life balance for healthcare workers, both the patients’ health outcomes and the HCW performance improve, also reducing the degree of burnout for the latter (5, 6).

There is evidence that, among HCW, physicians with a proper level of job satisfaction provide a high-quality service that better meets the health needs of patients, and that a higher risk of medical errors is linked to burnout and significant levels of stress among physicians (7–9).

Sustained stress can result in exhaustion, psychological and/or physical discomfort. There is evidence of a significant prevalence of burnout among practicing physicians (10, 11); according to the American Medical Association, the physician burnout rate in 2024 has dropped compared to 2022 (49% vs. 53%) and is similar to the 2023 rate (48.5%), after reaching substantial peaks during the COVID-19 pandemic (62.8% in 2021). Nevertheless, these statistics come from the USA, a country that uses ICD-10 (the version from which burnout syndrome is codified), while Italy still employs ICD-9, making the quantitative collection of affected people less rigorous and accurate, despite being a widely acknowledged issue for HCW (12).

Burnout syndrome stems from a variety of both individual and group factors, resulting in a particularly complex issue to address systematically (13). According to Patel et al., work-related factors that cause physicians’ burnout include excessive workloads, prolonged working hours, the type of residency chosen, having frequent on-call shifts (night or weekend), extensive paperwork duties, reduced free time due to work issues, risk of malpractice lawsuits, and coping with patients’ death and illness (14). Physicians consider loss of autonomy at work, decreased control over the work environment, the inefficient use of time due to administrative requirements, and loss of support from colleagues to be the main factors (15).

Emotional workplace stressors have proven to lead to negative consequences (i.e., higher rates of job abandonment, mental health-related disorders, inadequate strategies to manage stress in the absence of support) (16, 17) and have been exacerbated by the COVID-19 pandemic. Although technological progress in health care has enabled extraordinary progress in terms of prevention, health promotion, treatment and life expectancy in general (18, 19), it has also brought to light an ever-increasing and more complex amount of population health needs, which impose a greater workload on HCW. Universal healthcare systems are facing increasingly daunting challenges, directly impacting the personal and professional lives of physicians and dental surgeons (20, 21). Consequently, it is critical both to ensure an appropriate work-life balance and to provide the tools to pursue a satisfying and fulfilling career. The Physicians and Dental Surgeons Board of Rome and Province (OMCeO Rome) focuses on working conditions, professional and career development, and remuneration/incentives of its members, which is critical to sustaining the continuity of quality health care. Therefore, this study aimed to explore the work-life balance among OMCeO Rome members through a survey on their level of professional well-being and quality of life.

## 2 Materials and methods

### 2.1 Study design

A cross-sectional observational study was conducted from March 23rd, 2023, to June 9th 2023. An anonymous questionnaire was developed by a focus group promoted by the OMCeO Rome and proposed to all medical doctors and dental surgeons registered in the Province of Rome, Italy. The questionnaire consisted of 24 common questions for all profiles (4 profiles), and 3–9 specific questions for each profile (see Supplementary material), written in Italian and digitally designed through the logic of conditional branching of questions. The survey was uploaded on the OMCeO Rome institutional website to provide a rapid and easy access to all users interested, and a newsletter invitation to fill out the questionnaire was sent to all the members, followed by a few periodic reminders.

### 2.2 Measures

#### 2.2.1 Sociodemographic data

In the first section of the survey, respondents were asked to provide information on age, gender, marital status, potential family caregiver role. Moreover, information on professional activity (including specialization field and type of employment contract), years of experience, commute length/workplace distance from home, net yearly income (thousands of Euros/year), and weekly working hours were retrieved.

#### 2.2.2 Outcomes of interest

Several variables were used as outcome of interest, mostly related to work-life balance or perceived stress and health status. Such outcomes were collected using a Likert scale (1–5). Specifically, information was asked on:

- Perceived health status;
- Perceived global stress;
- Professional satisfaction;
- Satisfaction in relationships with peers/colleagues;
- Economic satisfaction;
- Intention to quit;
- Overall satisfaction.

### 2.3 Statistical analysis

Descriptive analysis was carried out, reporting mean values for continuous data and percentages for qualitative data. Chi^2^ test was performed to highlight differences in proportions by grouping variables in univariate analysis. A correlation analysis was used to investigate for collinearity among potentially explanatory variables, using Pearson’s r; threshold for collinearity was set at |r| ≥ 0.25. The statistical significance level was set at α = 0.05 for all analyses. The overall self-reported satisfaction level distribution was investigated via histogram and normal Q-Q plot. The 5-points Likert scale resulted in a very polarized distribution, with most respondents picking either side of the scale, and very few central values. Thus, this outcome was dichotomized, considering the upper values (3 and 4) as “High satisfaction,” and the lower values (0, 1, and 2) as “low satisfaction.” Those who answered neutrally (2 on the Likert scale) were considered as “low satisfaction” as well, since absence of satisfaction was still declared. A logistic model was then proposed, to investigate determinants of high and low self-reported overall satisfaction level. All non-collinear categorical variables with a consistent role in univariate analysis, plus gender and age class, were then used as candidate predictors in the final multivariable logistic model. Statistical analysis was performed using Stata v.17.0.

## 3 Results

Table 1 reports the sample characteristics, including socio-demographic and professional categories of the respondents, sorted by type of employment contract. The survey was answered by 1.104 doctors (in training and specialists) and dental surgeons (42.6% male, 56.6% female), all members of OMCeO Rome. Over half of the respondents were aged 50 or over, with 27.7% between 50 and 60, and 34.9% over 60. Only 3% were younger than 30, with the rest equally balanced in the ranges 20–30 (16.0%) and 30–40 (18.1%). Across each type of employment category, the most frequently reported marital status was “married/cohabiting,” except for the Residents, mostly reporting “single,” as it was also the youngest group. About 75% of the respondents declared a workplace distance within 20 km, in all categories. 45.3% of the respondents has a yearly income between €36.000 and €70.000, and no respondents earn less than €36.000; also, about 10% declared an income above €100.000 per year. No respondents declared less than 30 weekly working hours, and 38% of them works more than 40 h per week; lastly, 12.2% works more than 50.

**Table 1:** Socio-demographic and professional categories of the respondents, by type of employment.

	GP/PCP	Employed	Self-employed	Resident	Other	Total
Gender (Chi^2^ = 39.8*)						
Female	183 (59,4%)	277 (57,8%)	113 (48,1%)	48 (65,8%)	3 (37,5%)	625 (56,6%)
Male	123 (39,9%)	198 (41,3%)	121 (51,5%)	23 (31,5%)	5 (62,5%)	470 (42,6%)
NA**	2 (0,6%)	4 (0,8%)	1 (0,4%)	2 (2,7%)	0 (0,0%)	9 (0,8%)
Total	308 (100%)	479 (100%)	235 (100%)	73 (100%)	8 (100%)	1.104 (100%)
Marital status (Chi^2^ = 98.7*)						
Divorced/Separated	44 (14,3%)	57 (11,9%)	23 (9,8%)	0 (0%)	0 (0%)	124 (11,2%)
Single	45 (14,6%)	102 (21,3%)	48 (20,4%)	46 (63%)	2 (25%)	243 (22%)
Married/Cohabiting	213 (69,2%)	312 (65,1%)	150 (63,8%)	27 (37%)	5 (62,5%)	718 (65%)
Widowed	6 (1,9%)	8 (1,7%)	4 (1,7%)	0 (0%)	1 (12,5%)	19 (1,7%)
Total	308 (100%)	479 (100%)	235 (100%)	73 (100%)	8 (100%)	1.104 (100%)
Age (Chi^2^ = 521.7*)						
< 30	3 (1,0%)	1 (0,2%)	3 (1,3%)	29 (39,7%)	0 (0,0%)	36 (11,7%)
30 – 40	24 (7,8%)	70 (14,6%)	40 (17,0%)	40 (54,8%)	3 (37,5%)	177 (16,0%)
40 – 50	39 (12,7%)	125 (26,1%)	33 (14,0%)	3 (4,1%)	0 (0,0%)	200 (18,1%)
50 - 60	87 (28,2%)	162 (33,8%)	54 (23,0%)	1 (1,4%)	1 (12,5%)	306 (27,7%)
> 60	155 (50,3%)	121 (25,3%)	105 (44,7%)	0 (0,0%)	4 (50,0%)	385 (34,9%)
Total	308 (100%)	479 (100%)	235 (100%)	73 (100%)	8 (100%)	1.104 (100%)
Workplace distance (Chi^2^ = 82.1*)						
<5km	127 (41,2%)	102 (21,3%)	79 (33,6%)	19 (26,0%)	2 (25%)	329 (29,8%)
5-20km	124 (40,3%)	229 (47,8%)	105 (44,7%)	43 (58,9%)	5 (62,5%)	506 (45,8%)
20-40km	44 (14,3%)	87 (18,2%)	32 (13,6%)	8 (11,0%)	1 (12,5%)	172 (15,6%)
>40km	13 (4,2%)	61 (12,7%)	19 (8,1%)	3 (4,1%)	0 (0,0%)	97 (8,8%)
Total	308 (100%)	479 (100%)	235 (100%)	73 (100%)	8 (100%)	1.104 (100%)
Income/year (thousands) (Chi^2^ = 83.7*)						
<€36	0 (0,0%)	0 (0,0%)	0 (0,0%)	0 (0,0%)	0 (0,0%)	0 (0,0%)
€36-70	128 (41,6%)	259 (54,1%)	107 (45,5%)	4 (5,5%)	2 (25%)	500 (45,3%)
€70-100	102 (33,1%)	148 (30,9%)	41 (17,4%)	0 (0,0%)	2 (25%)	293 (26,5%)
> €100	46 (14,9%)	27 (5,6%)	17 (7,2%)	0 (0,0%)	0 (0,0%)	90 (8,2%)
*Missing*	*32 (10,4%)*	*45 (9,4%)*	*70 (29,8%)*	*69 (94,5%)*	*4 (50,0%)*	*221 (20,0%)*
Total	308 (100%)	479 (100%)	235 (100%)	73 (100%)	8 (100%)	1.104 (100%)
W-H***/week (Chi^2^ = 70.6*)						
<30	0 (0,0%)	0 (0,0%)	0 (0,0%)	0 (0,0%)	0 (0,0%)	0 (0,0%)
30-40	152 (49,4%)	217 (45,3%)	75 (31,9%)	19 (26,0%)	5 (62,5%)	468 (42,4%)
40-50	73 (23,7%)	212 (44,3%)	46 (19,6%)	24 (32,9%)	1 (12,5%)	356 (32,2%)
>50	29 (9,4%)	44 (9,2%)	14 (6,0%)	27 (37,0%)	0 (0,0%)	114 (10,3%)
*Missing*	*54 (17,5%)*	*6 (1,3%)*	*100 (42,6%)*	*3 (4,1%)*	*2 (25,0%)*	*166 (15,0%)*
Total	308 (100%)	479 (100%)	235 (100%)	73 (100%)	8 (100%)	1.104 (100%)
Night shifts (Chi^2^ = 183.2*)						
No	284 (92,2%)	189 (39,5%)	205 (87,2%)	28 (38,4%)	7 (87,5%)	714 (64,7%)
Yes	24 (7,8%)	290 (60,5%)	30 (12,8%)	45 (61,6%)	1 (12,5%)	390 (35,3%)
Total	308 (100%)	479 (100%)	235 (100%)	73 (100%)	8 (100%)	1104 (100%)

*p < 0.001. **not answered, ***W-H/week: working hours per week.

The General Practitioners (GP)/Primary Care Paediatricians (PCP) group is mostly female, half are more than 60 years old, living within 20 km from their workplace, with a yearly income between €36.000 and €70.000 and declare 30 to 40 working hours per week, mostly without night shifts.

The National Health System (NHS) employees’ group is 57.8% female, 40 to 60 years old, living within 5 to 20 km from their workplace, has a yearly income between €36.000 and €70.000, 30 to 50 working hours per week, and with night shifts in 60.5% of cases.

The self-employed group’s gender is mixed (about 50% for each gender), 60 years old, with a workplace distance between 5 and 20 km, a yearly income between €36.000 and €70.000, 30 to 50 working hours per week, without night shifts.

Lastly, the resident’s group (which includes clinicians, surgeons, and clinical services specialists) is generally female, 30 to 40 years old, with a workplace distance between 5 and 20 km, a yearly income not declared in most cases (residents have a fixed economic treatment in Italy), lower than €36.000; more than one third of residents declare to work more than 50 h per week, with night shifts in most cases.

**Figure 1:**
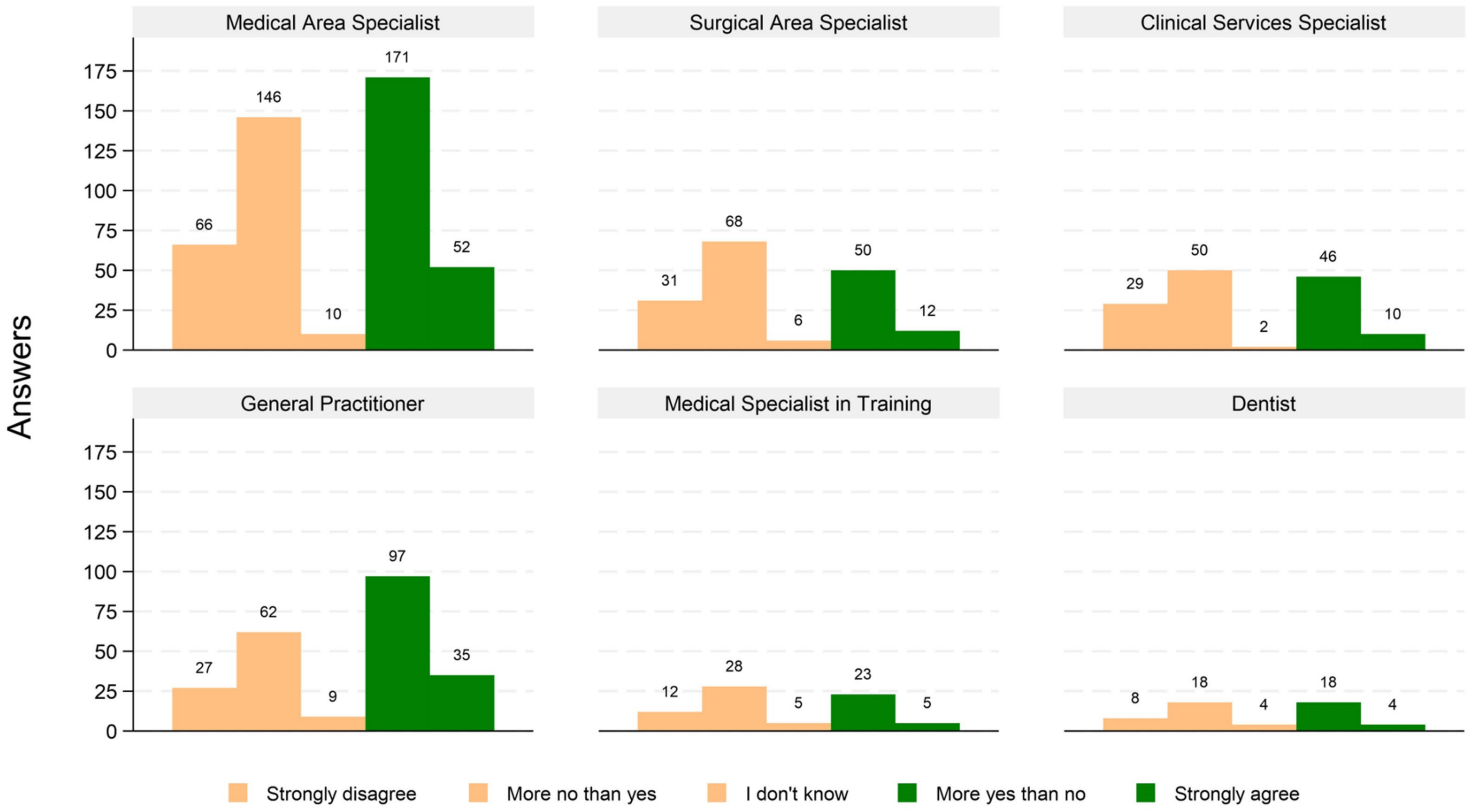
Self-reported overall satisfaction levels, divided by professional category.

Figure 1 shows the self-reported overall satisfaction levels, by professional category. The answers distribution regarding overall satisfaction levels is polarized, as 523 (47.4%) reported high level of satisfaction, 49.4% low level of satisfaction, and only 3.3% answered neutrally, a pattern that is consistent throughout all professional categories.

**Figure 2:**
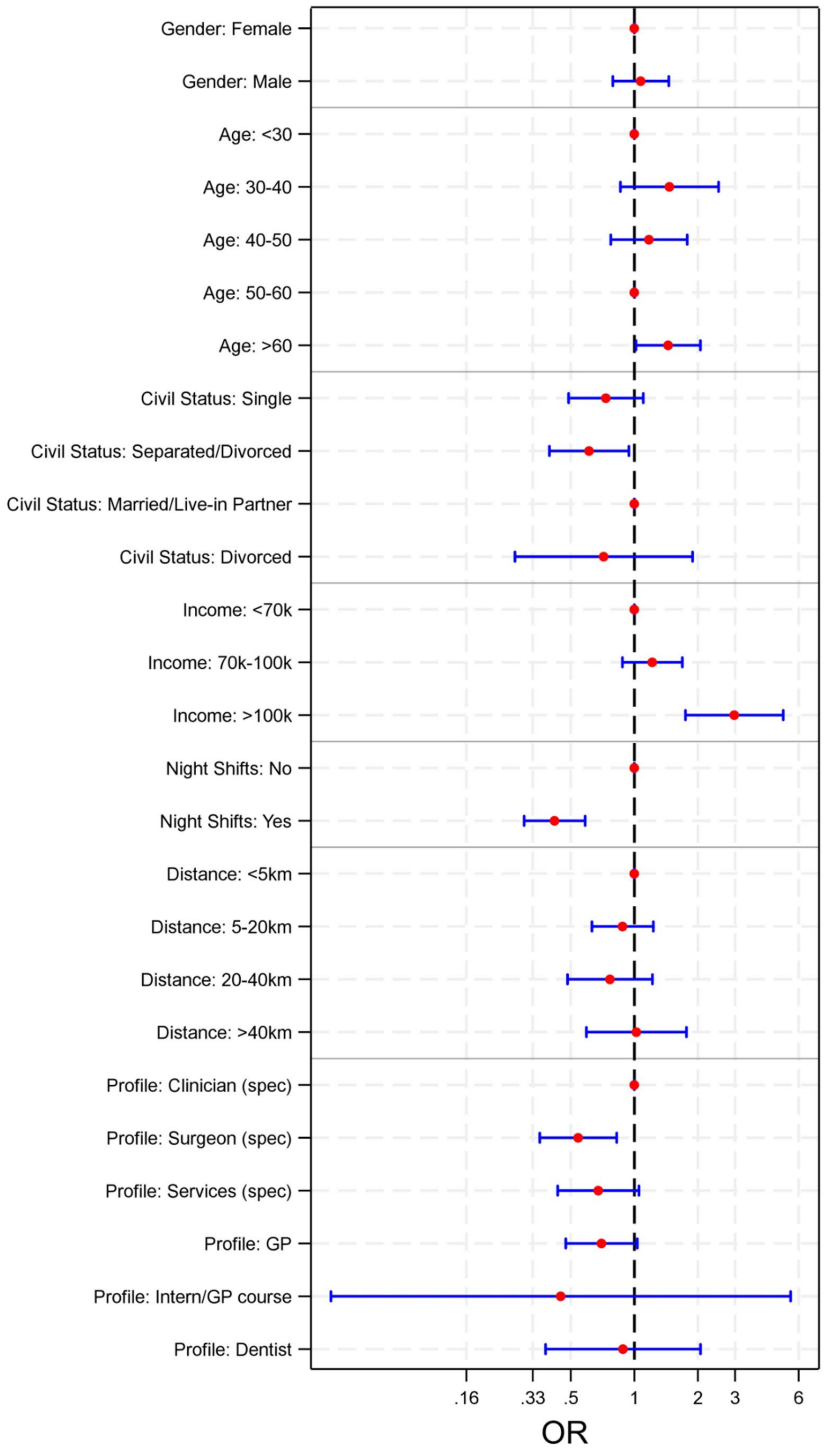
Forest plot for the multivariable logistic regression.

The univariable logistic analysis in Supplementary Table S1 shows that the self-employed group has reported less frequently work-related stress (OR 0.31 [95% CI 0.19–0.52]), thought of quitting work (OR 0.50 [95% CI 0.33–0.76]), and dissatisfaction (career-wise (OR 0.39 [95% CI 0.27–0.59]) and economic-wise (OR 0.25 [95% CI 0.16–0.41])) compared to the other groups. Males are more prone to declare economic dissatisfaction (OR 1.45 [95% CI 1.01–2.10]) compared to females, but also less prone to declare poor/very poor perceived health (OR 0.50 [95% CI 0.37–0.66]). Across all groups, the GP/PCP group has reported more often high levels of work-related stress (OR 1.45 [95%CI 1.01–2.07]). Moreover, having to work night shifts has consistently shown association with thoughts of quitting work (OR 1.14 [95% CI 1.02–2.08]), higher work-related stress (OR 1.57 [95% CI 0.81–1.52]), and dissatisfaction career-wise (OR 1.70 [95% CI 1.25–2-32]) and economic-wise (OR 2.23 [95% CI 1.36–3.67]) among responders. Lastly, living alone has shown association with thinking often of quitting work (OR 1.59 [95%CI 1.04–2.41]), and being a caregiver is associated with high work-related stress perceived (OR 1.39 [95%CI 1.02–1.89]). A multivariable logistic regression was performed to study the determinants for overall satisfaction, and the forest plot is shown in Figure 2. The most significant elements leading to low satisfaction were represented by night shifts schedules and income levels. Those whose job did not involve night shifts were twice as likely to report higher satisfaction levels (OR 1.9, *p* < 0.001). Income levels were a good predictor only for the upper income range (more than €100.000 /year; OR: 3.9, *p* < 0.001). Other socio-economic and professional elements were not significantly impactful on global satisfaction.

## 4 Discussion

The present study surveyed a group of physicians, members of OMCeO Rome, aiming to analyze their socio-demographic and professional characteristics and shed light on the factors influencing their work-life balance and job satisfaction. The answers gathered have revealed different age groups, mostly composed by females, and significant variety regarding income and working conditions across employment types. Overall satisfaction was polarized, with nearly half expressing high satisfaction and a similar proportion reporting dissatisfaction, influenced by night shifts and income levels. The self-employed group reported lower work-related stress and dissatisfaction, while GPs/PCPs and those working night shifts or living alone experienced higher stress and thoughts of quitting. The respondents were mostly adult, non-surgeon specialists, with a middle income and workplace distance. This analysis also underlines the crucial role played by the high number of working hours and night shifts of residents, in contrast to the low economic incentives, which determined low overall satisfaction with their work-life balance. A poor working life quality stems from multiple causes, attributable both to direct experiences in the workplace (i.e., lack of adequate resources, excessive workload, high professional risk, low compensation), and indirect ones, in private life (i.e., symptoms related to stress and anxiety, sleep disorders, family tensions). Both may lead to burnout syndrome, which can cause abandonment of the profession, greater recurrence of errors during medical practice, and reduced overall quality of care (22).

Work-related stress is a widely studied phenomenon in the international scientific literature. In a German study on GP, about one third felt emotionally exhausted and depersonalized, regardless of the few night shifts worked (23). Other studies have proven a beneficial association between a reasonable work-life balance and low work-related stress, as well as less likely burnout onset and more professional motivation (24–26). Our results are in line with other studies that show connections between working overtime, and/or working night shifts, and high work-related stress in medical professionals. Hence, administrative, legal and bureaucratic supports as well as working fewer hours could help mitigate this phenomenon. Beyond benefitting GPs and their patients, this could also help to reduce costs in the healthcare system that are caused by physician turnover (27–29). The study presents several strengths and limitations. As is inherent in cross-sectional studies that include a questionnaire, the data are self-reported; thus, individuals may offer biased estimates, since respondents to a questionnaire are more likely to be most interested and/or involved in the topic than others. Moreover, the design of the study does not provide insight into time-dependent aspects of how factors associated with respondents’ job satisfaction may impact them over time, nor it allows to study causality, beyond associations. More than half of the respondents were 50 or older; a higher number of younger respondents could influence the results differently. Other dimensions such as the mix-case, complexity of patients, mental health status of physicians. Our results do not provide direction on whether systemic or individual interventions would be effective in decreasing physician burnout. Nonetheless, the accessibility of the survey has brought forth a representative sample size; the statistical analysis has followed a rigorous methodology, and the relevance of the factors explored has been carefully considered. Further analyses of this survey could investigate whether socio-cultural and individual factors may have a cross-cutting effect (e.g., gender and years of practice); moreover, by addressing these influences on work-related stress, insights for targeted policies and interventions could be drawn. Moreover, a qualitative research design could provide more detailed insights on the emotional components of daily working conditions of physicians. Lastly, in order to explore more precisely the role that perceived stress caused by work plays in work-life balance, the respondent’s expectations on the latter with respect to their chosen career type could be investigated further.

In conclusion, the findings of this study highlight the complex interplay between professional and personal factors influencing physicians’ work-life balance and job satisfaction. The results underscore the need for targeted interventions to address excessive workloads, especially night shifts, insufficient compensation, and the broader implications of occupational stress on both physician well-being and the quality of patient care. Ensuring HCW a satisfying work-life balance relies on training, up-to-date information, and proactive leadership, to help professionals better manage emotional challenges and building trustful, healthy workplaces that both incentivize professionals to stay and aspirants to approach the profession.

## Data Availability

The raw data supporting the conclusions of this article will be made available by the authors, without undue reservation.

## Group members of Rome OMCeO Group

Rome OMCeO Group members: Antonio Magi, Stefano De Lillo and Guido Coen Tirelli.
