# Supplementary material for: Factors associated with satisfaction of Italian physicians: a cross-sectional study in Rome
Source: Front Public Health. 2025 May 30;13:1584483. doi: 10.3389/fpubh.2025.1584483 (PMC12162915; doi:10.3389/fpubh.2025.1584483)
Supplement: Supplementary file 1 [file Table_1.DOCX]

| ***Age (years)*** | **Have you thought of quitting work?** | | | | **High work-related stress perceived**  OR (95% CI) p | | **Poor/very poor perceived health**  OR (95% CI) p | | **Dissatisfaction** | | | |
| --- | --- | --- | --- | --- | --- | --- | --- | --- | --- | --- | --- | --- |
|  | **Often**  OR (95% CI) | p | **Often/Sometimes**  OR (95% CI) p | |  |  |  |  | **Career**  OR (95% CI) | p | **Economic**  OR (95% CI) | p |
| <30 | 1 | | 1 | | 1 | | 1 | | 1 | | 1 | |
| 30-40 | 0.89 (0.37-2.14) | 0.794 | 1.89 (0.78-4.55) | 0.157 | 1.61 (0.63-4.13) | 0.323 | 0.69 (0.27-1.77) | 0.443 | 1.32 (0.57-3.05) | 0.517 | 2.17 (0.61-7.74) | 0.230 |
| 40-50 | 0.86 (0.33-2.24) | 0.763 | 1.42 (0.53-3.79) | 0.483 | 2.21 (0.79-6.20) | 0.132 | 0.95 (0.34-2.63) | 0.922 | 1.19 (0.47-2.98) | 0.712 | 1.13 (0.30-4.32) | 0.855 |
| 50-60 | 0.84 (0.32-2.16) | 0.711 | 1.40 (0.53-3.72) | 0.494 | 2.24 (0.80-6.26) | 0.123 | 0.97 (0.35-2.67) | 0.955 | 1.36 (0.55-3.39) | 0.508 | 1.08 (0.29-4.08) | 0.910 |
| >60 | 0.67 (0.26-1.75) | 0.418 | 0.95 (0.36-2.49) | 0.912 | 1.78 (0.63-4.99) | 0.275 | 1.10 (0.40-3.04) | 0.849 | 1.04 (0.42-2.60) | 0.929 | 1.06 (0.28-4.02) | 0.926 |
| ***Type of employment contract*** |  | |  | |  | |  | |  | |  | |
| Employed | 1 | | 1 | | 1 | | 1 | | 1 | | 1 | |
| **GP/PCP** | 0.77 (0.54-1.10) | 0.154 | 0.91 (0.62-1.34) | 0.627 | **1.45 (1.01-2.07)** | **0.043** | 0.98 (0.68-1.42) | 0.930 | 0.73 (0.52-1.03) | 0.072 | 0.78 (0.48-1.28) | 0.332 |
| **Self-employed** | **0.50 (0.33-0.76)** | **0.001** | **0.36 (0.24-0.54)** | **<0.001** | **0.31 (0.19-0.52)** | **<0.001** | 0.91 (0.60-1.39) | 0.682 | **0.39 (0.27-0.59)** | **<0.001** | **0.25 (0.16-0.41)** | **<0.001** |
| Resident | 0.56 (0.28-1.14) | 0.110 | 0.50 (0.24-1.04) | 0.062 | 1.40 (0.69-2.83) | 0.354 | 0.85 (0.40-1.84) | 0.686 | 0.91 (0.47-1.76) | 0.777 | 1.14 (0.36-3.62) | 0.825 |
| **Dentist** | 0.92 (0.49-1.74) | 0.810 | 0.91 (0.45-1.85) | 0.803 | 0.67 (0.32-1.40) | 0.291 | 1.11 (0.56-2.19) | 0.756 | 0.89 (0.48-1.65) | 0.706 | **0.33 (0.16-0.68)** | **0.003** |
| ***Gender*** |  | |  | |  | |  | |  | |  | |
| F | 1 | | 1 | | 1 | | 1 | | 1 | | 1 | |
| M | 0.87 (0.67-1.14) | 0.329 | 0.96 (0.71-1.28) | 0.762 | 1.16 (0.88-1.54) | 0.290 | **0.50 (0.37-0.66)** | **<0.001** | 0.95 (0.73-1.23) | 0.680 | **1.45 (1.01-2.10)** | **0.046** |
| ***Night shifts*** |  | |  | |  | |  | |  | |  | |
| No | 1 | | 1 | | 1 | | 1 | | 1 | | 1 | |
| **Yes** | 1.11 (0.81-1.52) 0.040 | | **1.46 (1.02-2.08)** | **0.040** | **1.57 (1.13-2.19)** | **0.007** | 1.04 (0.75-1.46) | 0.796 | **1.70 (1.25-2.32)** | **0.001** | **2.23 (1.36-3.67)** | **0.002** |
| ***Workplace distance (km)*** |  | |  | |  | |  | |  | |  | |
| < 5 | 1 | | 1 | | 1 | | 1 | | 1 | | 1 | |
| 5-20 | 1.01 (0.74-1.37) | 0.953 | 1.10 (0.79-1.53) | 0.561 | 0.89 (0.65-1.23) | 0.494 | 0.82 (0.59-1.12) | 0.210 | 1.21 (0.89-1.63) | 0.216 | 1.22 (0.83-1.81) | 0.315 |
| 20-40 | 0.96 (0.64-1.44) | 0.839 | 1.23 (0.78-1.94) | 0.363 | 1.16 (0.76-1.76) | 0.487 | 1.08 (0.72-1.64) | 0.706 | 1.34 (0.90-1.99) | 0.151 | 1.46 (0.82-2.58) | 0.195 |
| >40 | 1.02 (0.62-1.66) | 0.840 | 0.87 (0.51-1.48) | 0.610 | 1.29 (0.78-2.14) | 0.319 | 0.81 (0.48-1.36) | 0.432 | 0.96 (0.59-1.56) | 0.871 | 1.20 (0.61-2.37) | 0.589 |
| ***Living alone*** |  | |  | |  | |  | |  | |  | |
| No | 1 | | 1 | | 1 | | 1 | | 1 | | 1 | |
| **Yes** | **1.59 (1.04-2.41)** | **0.030** | 1.53 (0.96-2.43) | 0.075 | 0.85 (0.55-1.32) | 0.475 | 1.22 (0.79-1.87) | 0.370 | 1.30 (0.86-1.96) | 0.212 | 1.40 (0.78-2.52) | 0.257 |
| ***Marital status*** |  | |  | |  | |  | |  | |  | |
| Married/Living with partner | 1 | | 1 | | 1 | | 1 | | 1 | | 1 | |
| Not married | 0.82 (0.54-1.24) | 0.346 | 0.73 (0.47-1.14) | 0.167 | 1.43 (0.94-2.19) | 0.097 | 1.09 (0.71-1.67) | 0.686 | 1.14 (0.76-1.70) | 0.521 | 0.70 (0.41-1.20) | 0.197 |
| Separated/Divorced | 0.91 (0.58-1.42) | 0.677 | 1.03 (0.63-1.69) | 0.893 | 1.46 (0.93-2.29) | 0.099 | 1.32 (0.85-2.06) | 0.215 | 1.52 (0.98-2.36) | 0.060 | 1.25 (0.66-2.34) | 0.490 |
| Widow/Widower | 0.47 (0.14-1.51) | 0.204 | 0.48 (0.17-1.33) | 0.158 | 2.73 (0.97-7.65) | 0.056 | 0.97 (0.34-2.75) | 0.949 | 0.95 (0.35-2.60) | 0.927 | 1.00 (0.26-3.82) | 0.999 |
| ***Being a caregiver*** |  | |  | |  | |  | |  | |  | |
| No | 1 | | 1 | | 1 | | 1 | | 1 | | 1 | |
| **Yes** | 1.11 (0.83-1.48) | 0.492 | 1.05 (0.77-1.43) | 0.745 | **1.39 (1.02-1.89)** | **0.035** | 1.34 (0.99-1.82) | 0.058 | 1.24 (0.93-1.65) | 0.133 | 1.31 (0.90-1.90) | 0.164 |

**Table S1.** Univariate analysis results. In bold, factors that have shown a significant association (p-value <0.05) with the questions asked in the survey.

**Abbreviations** OR: Odds Ratio. GP/PCP: General Practitioner/Primary Care Paediatrician. P: p-value (< 0.05). F: Female. M: Male.
